# Central Dental Association of Northern New Jersey

**Published:** 1887-11

**Authors:** 


### CENTRAL DENTAL ASSOCIATION OF NORTHERN NEW JERSEY.

REPORTED FOR THE INDEPENDENT PRACTITIONER.

A paper by Dr. Wm. H. Trueman, entitled "The Tooth Pulp in its Relation to Dental Operations," was read by Dr. Luckey. (See page 569.)

*Dr. R. B. Winder*—I would be loth to assent to the proposition which the essayist seems to have stated very earnestly, that the death of a part of the pulp necessarily involves the death of the whole. That is against all physiological research. You might as well claim that the death of the hand would involve the death of the arm. The pulp, being a delicate tissue, is, under the influence of disease, liable to die, but it does not follow as a matter of necessity that it should do so in every case. I suspect there are gentlemen here present who have in their practice seen instances, particularly in the lower molar teeth, where it has been found necessary to excise the anterior or the posterior root, and the pulp remained alive in the one that was left.

*Dr. Atkinson*—I am very much in accord with what Prof. Winder has said. I learned that the remnant of a pulp that had been taken out for one-half or three-fourths of its length, in a single root upper tooth, was capable of healing and remaining for twelve or fifteen years in a perfectly healthy condition. That was taught me by experience. I have repeatedly taken portions of well elaborated pus from the cornua of the pulp of lower molars especially, and I have had the pulp heal and remain kindly as long as I kept the history of the case in my mind.

I had a case of a young lady who was preparing for the stage, who came to me suffering with that intense bounding pain which is almost unbearable. There was periostitis as well as pulpitis present. I had to secure a ligature around the neck of the tooth upon which to make traction, to make it possible for her to bear the operation of opening into the pulp chamber with a very fine drill. I drilled directly through the remaining portion of the dentine that was not decayed, so as to expose the pulp, and pus exuded upon the withdrawal of the drill, and subsequently fresh blood flowed out, indicating the limitation of the abscess with the cornua of the pulp. After two or three days' treatment it healed kindly, and I filled with oxy-phosphate of zinc. That was about twelve years since.

That brings up another little matter of experience. When you are using the oxy-chlorides, or the chloride of zinc solution pretty strong, you will sometimes get some of it down in the tooth when it has not been properly treated before with an obtunder, and in some of those cases I have found the pulp to have been converted into a chloro-zincate of albumen, as I call it. In extracting some of those teeth afterwards, I found that some of the pulps had been converted into this glass-like appearance, leading me to put a very high estimate upon the presence of the chloride of zinc, which had so completely disinfected the neighborhood that it prevented any subsequent periostitis

